# Occupational Exposure to Hexavalent Chromium, Nickel and PAHs: A Mixtures Risk Assessment Approach Based on Literature Exposure Data from European Countries

**DOI:** 10.3390/toxics10080431

**Published:** 2022-07-29

**Authors:** Ana Maria Tavares, Susana Viegas, Henriqueta Louro, Thomas Göen, Tiina Santonen, Mirjam Luijten, Andreas Kortenkamp, Maria João Silva

**Affiliations:** 1 Departamento de Genética Humana, Instituto Nacional de Saúde Dr. Ricardo Jorge (INSA), Avenida Padre Cruz, 1649-016 Lisbon, Portugal; anamptavares@gmail.com (A.M.T.); henriqueta.louro@insa.min-saude.pt (H.L.); 2 ToxOmics–Centre for Toxicogenomics and Human Health, NOVA Medical School, Universidade NOVA de Lisboa, Campo dos Mártires da Pátria, 130, 1169-056 Lisbon, Portugal; 3 Public Health Research Centre, NOVA National School of Public Health, Universidade NOVA de Lisboa, 1600-560 Lisbon, Portugal; susana.viegas@ensp.unl.pt; 4 Comprehensive Health Research Center (CHRC), 1169-056 Lisbon, Portugal; 5 Institute of Occupational, Social and Environmental Medicine (IPASUM), University Erlangen-Nürnberg, Henkestraße 9-11, 91054 Erlangen, Germany; thomas.goeen@fau.de; 6 Finnish Institute of Occupational Health, FI-00250 Helsinki, Finland; tiina.santonen@ttl.fi; 7 Centre for Health Protection, National Institute for Public Health and the Environment, 3720 BA Bilthoven, The Netherlands; mirjam.luijten@rivm.nl; 8 Centre for Pollution Research and Policy, College of Health, Medicine and Life Sciences, Brunel University London, Kingston Lane, Uxbridge, London UB8 3PH, UK; andreas.kortenkamp@brunel.ac.uk

**Keywords:** human biomonitoring (HBM), European Human Biomonitoring Initiative (HBM4EU), co-exposure, workplace, mixture risk assessment (MRA)

## Abstract

Hexavalent chromium (Cr(VI)), nickel (Ni) and polycyclic aromatic hydrocarbons (PAHs) are genotoxic co-occurring lung carcinogens whose occupational health risk is still understudied. This study, conducted within the European Human Biomonitoring Initiative (HBM4EU), aimed at performing a mixtures risk assessment (MRA) based on published human biomonitoring (HBM) data from Cr(VI), Ni and/or PAHs occupational co-exposure in Europe. After data extraction, Risk Quotient (RQ) and Sum of Risk Quotients (SRQ) were calculated for binary and ternary mixtures to characterise the risk. Most selected articles measured urinary levels of Cr and Ni and a SRQ > 1 was obtained for co-exposure levels in welding activities, showing that there is concern regarding co-exposure to these substances. Similarly, co-exposure to mixtures of Cr(VI), Ni and PAHs in waste incineration settings resulted in SRQ > 1. In some studies, a low risk was estimated based on the single substances’ exposure level (RQ < 1), but the mixture was considered of concern (SRQ > 1), highlighting the relevance of considering exposure to the mixture rather than to its single components. Overall, this study points out the need of using a MRA based on HBM data as a more realistic approach to assess and manage the risk at the workplace, in order to protect workers’ health.

## 1. Introduction

Industrial activities such as metal processing, electroplating, chromate production, welding, and thermal cutting make use of chromium and represent the main sources of hexavalent chromium (Cr(VI)) occupational exposure [1,2]. Chromium exposure occurs mainly by inhalation, making the lungs the main target organ for its effects [1]. The occupational exposure to Cr(VI) has been associated with nasal and sinus cancer and with lung, trachea, and bronchus cancers [2]. Cr(VI) compounds have been classified by IARC as carcinogenic to humans (group 1) [3]. The induction of DNA strand breaks, inter-strand cross-links and DNA adducts are deemed to mediate Cr(VI) genotoxicity [1]. This effect is also due to the contribution of oxidative damage originated from reactive oxygen species (ROS) that arise from a series of intracellular Cr(VI) reduction steps. Cytogenetic alterations, such as chromosomal breaks and micronuclei (MN) have also been reported and suggested to be the outcome of double-strand breaks induction [3].

Nickel has been widely used in stainless steel manufacturing, electroplating, foundry applications, printing inks, and prostheses manufacturing, which causes internal levels of Ni significantly to be higher among exposed workers [1]. Exposure by inhalation, ingestion or skin contact can occur in nickel industries [4]. Occupationally inhaled Ni is typically associated with immunological sensitisation, epithelial dysplasia and asthma, and can also cause nasal and lung fibrosis and cancer [1,5,6]. Similarly to Cr(VI), Ni was classified by IARC as carcinogenic to humans [4]. Its genotoxic effects include formation of DNA strand breaks, DNA cross-links and chromosomal aberrations. Both Cr(VI) and Ni can also interfere with DNA repair mechanisms, enhancing the genotoxic effect of other agents [1,7].

Polycyclic aromatic hydrocarbons (PAHs) are widespread environmental contaminants continuously formed during incomplete combustion or pyrolysis of organic material. Their major environmental sources are heating, motor-vehicle exhaust and industrial emissions such as industrial power generation, incinerators, the production of coal tar, coke and asphalt, and petroleum catalytic cracking [8,9]. Recent technology developments have also been using pyrolysis for the production of biofuel from microalgal biomass. However, under inappropriate conditions, this can also result in the emission of PAHs [10,11]. Occupational exposure to PAHs may occur by breathing exhaust fumes, by ingestion of contaminated food and by dermal absorption due to handling of contaminated materials and surfaces. High levels of pollutant mixtures containing PAHs can cause irritation, inflammation, nausea, and vomiting. A long exposure may cause an increased risk of skin and lung cancer. Similarly to Cr(VI) and Ni, PAHs such as benzo(a)pyrene (BaP) can cause ROS-related but also PAH-specific DNA adducts, and chromosomal alterations, reflected in the induction of chromosomal breaks, sister chromatic exchanges (SCEs) and MN [12,13]. Although IARC classifications vary across the existing PAHs, with many of them classified as probably and possibly carcinogenic to humans (group 2A and 2B, respectively) [14], BaP is so far the only one classified as carcinogenic to humans [15].

Occupational co-exposure to Cr(VI) and Ni has been well documented in the literature, due to its frequent occurrence in the same workplace, namely, during welding operations [16,17]. Moreover, a study in Finland demonstrated that Cr, Ni, BaP and other PAHs were amongst the main substances to which welders and flame cutters were co-exposed between 2007 and 2009 [18]. Workers from aluminium production, iron and steel founding industries, waste incineration sites and coke oven plants can be also occupationally co-exposed to PAHs and to metals including Cr and Ni [19,20]. In addition, it has been demonstrated that workers performing painting activities can be highly co-exposed to mixtures of Cr and PAHs as common paint components [19].

Despite the acknowledged evidence of co-exposure, the combined health effects caused by these three substances have been rarely investigated. Current regulatory practices are usually based on single chemical substances [21,22], without integrating the possibility co-exposure or aggregated exposure. The few existing in vitro and in vivo data suggest that combinations of Cr, Ni and PAHs can lead to adverse effect that are higher than those observed for each single substance [23,24,25]. The multiple chemicals involved in exposure scenarios may lead to myriad interactions with a wide array of underlying mechanisms. This may ultimately result in different health outcomes from those expected from the single substances [26]. In some cases, only one or few components may dominate the overall mixture hazard, and therefore, the identification of the main active components, i.e., the drivers of mixture toxicity, may allow the implementation of more efficient risk reduction measures [27]. Thus, failing to account for the combined effects of co-exposures can lead to an underestimation of the risk. In this sense, the recently published “Chemicals Strategy for Sustainability” claims the need for integrating mixtures risk assessment (MRA) more generally into chemical risk assessment in the scope of different regulatory frameworks [28].

**Figure 1:**
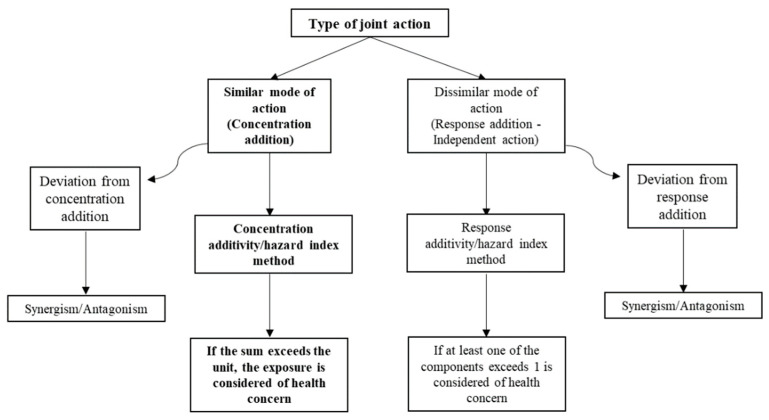
Flowchart representing the types of joint action (similar and dissimilar) and the respective applicable methods in mixtures risk assessment.

Approaches for a more holistic mixtures risk assessment (MRA) currently remain as research challenges [27]. Knowing the mode of action (MoA) of a substance is essential to perform an accurate MRA. In this regard, two main mathematical models can be used to assess the effects of chemical mixtures: one assumes that individual substances act via a similar MoA and applies dose or concentration addition (CA), while the other model assumes dissimilar MoAs (independent action (IA)) and applies response addition. Synergism and antagonism due to the interaction of substances in a mixture can be defined in relation to this additivity assumption. In this context, the term interaction (synergistic or antagonistic effects) includes all forms of joint actions that deviate from either dose or response addition (Figure 1) [29,30]. If a group of chemicals affect the same toxicological endpoint and/or have the same MoA, as in the case of Cr(VI), Ni and PAHs, it seems plausible to generate a mixtures risk indicator, which requires individual data [31,32,33]. However, the distinction between similar and dissimilar MoAs in real exposure scenarios with complex chemical mixtures is often unclear [29,34]. There is now a growing consensus that MRAs should start from the default assumption of CA for all mixture components, regardless of the MoAs. If this indicates a significant risk, refined MoA-based assessments may be conducted where the necessary data is available [34,35].

The use of human biomonitoring (HBM) data has been gaining momentum in identifying realistic co-exposure patterns in the same environmental or human samples [27]. Total Cr measurements in urine have been used as indicators of occupational exposure [36]; correlation has been also reported between air and urinary nickel levels in several occupational settings [37]; regarding PAHs, the urinary metabolite 1-hydroxypyrene (1-OHP) has been the most frequently analysed biomarker in occupational studies [38]. Besides measuring co-exposure, it is important to detect its early biological effects, and for that purpose, the combination of exposure data with effect biomarkers is essential to assess potential human health effects. Most of these biomarkers do not point to an individual cause, but they may reflect an integrated effect of an aggregated exposure [39]. Micronuclei in human peripheral blood lymphocytes, alkaline comet assay, and oxidative stress biomarkers have been used to assess the genotoxic and carcinogenic effects of several substances [40]. However, few occupational HBM studies present data on effect biomarkers and link them to exposure data.

In the European Human Biomonitoring Initiative (HBM4EU), several substances were identified as research priorities, including Cr(VI), PAHs, and chemical mixtures (https://www.hbm4eu.eu/about-hbm4eu/, accessed on 10 March 2022). Within the HBM4EU scope, a recent multicentric study on the occupational exposure to Cr(VI) has been conducted in a harmonised way in several European countries [40,41], and other field studies are currently underway to provide information on human exposure and co-exposure. This study, developed under the HBM4EU initiative, aimed to perform a MRA for the co-exposure to Cr(VI), Ni and/or PAHs in several occupational settings in Europe, based on external exposure and HBM data gathered from the literature. This work intended to contribute to the development of a consistent and uniform framework applicable to future studies on mixtures exposure and health outcomes.

## 2. Materials and Methods

### 2.1. Literature Search and Data Collection

The literature searches were conducted in PubMed^®^ database between May and June 2020, using combinations of key words and/or Medical Subject Headings (MeSH) terms to find articles presenting HBM measurements. Different search strings for the possible combinations (binary and ternary) of Cr(VI), Ni and/or PAHs in occupational settings were used and are detailed in Supplementary Table S1.

Our searches were filtered to retrieve articles published between 2000 and 2020. This time window allowed us to account for the most recent working conditions and technological developments and for new risk management measures, while considering the influence of the regulatory frameworks presently in place. Only peer-reviewed articles which had abstracts and were written in English, Spanish, French or Portuguese were considered. Books or book chapters, comments, editorials, reviews, guidelines, reports, newspaper articles and case studies were excluded from the analysis. Articles meeting the following inclusion criteria were selected: (i) studies presenting biomarkers of exposure for Cr(VI) + Ni, Cr(VI) + PAHs, Ni + PAHs, or Cr(VI) + Ni + PAHs; (ii) HBM data obtained within occupational settings; (iii) studies including data on effect biomarkers (studies with data for a single substance only, but presenting effect biomarkers were still considered); iv) studies performed in EU countries. The following exclusion criteria were also defined: (i) studies conducted outside the EU (UK was still considered); (ii) studies not presenting HBM data for Cr(VI), Ni and/or PAHs, or presenting only graphical data; (iii) studies conducted in non-occupational settings (e.g., environmental studies). Following articles’ retrieval, titles and abstracts were first screened, and the selected articles proceeded to full text analysis.

In a second approach, references cited in the retrieved articles were searched, and only those meeting the inclusion criteria and presenting biomarkers of exposure for the three substances were selected. Data on biomarkers of exposure, effect biomarkers (e.g., comet assay, micronucleus assay), occupational settings, and exposure scenarios were extracted from the selected articles, whenever described. All data extracted was presented as arithmetic means, geometric means or medians.

### 2.2. Determination of the Sum of Risk Quotients

The Sum of Risk Quotients (SRQ), also called Hazard Index (HI) [40], is a regulatory approach to component-based MRA derived from the CA reference model. The SRQ was calculated for each mixture, whenever possible, according to Equation (1):

(1) $$\mathrm{SRQ}=\overset{n}{\underset{i=1}{\sum }}\frac{{\mathrm{EL}}_{i}}{{\mathrm{AL}}_{i}}$$

The Risk Quotients (RQ), i.e., the ratio of a substance/metabolite concentration in a biological specimen (EL), and its acceptable level (AL), (EL_i_/AL_i_), were calculated using data from the selected studies [31,32,42].

Table 1 presents the tolerable risk levels for Cr(VI), Ni and BaP in air used for RQ and SRQ calculations. Because there is no consensus on the acceptable or tolerable risk levels in Europe for occupational activities involving carcinogenic substances, an expert ad hoc group met to debate this topic, and a decision on the use of the tolerable risk level of 4:1000, as defined in the German Technical Rules for Hazardous Substances produced by the Committee on Hazardous Substances from the Federal Institute for Occupational Safety and Health), was reached. An additional advantage was that the “Risk Concept for Carcinogenic Substances” of the AGS, which offers a benchmark for comparison and assessment of exposure to carcinogenic substances, included limit values (in air) for the three components of the mixture under study. It must be stated that these levels still contain a residual risk for cancer [43]. Calculations using Equation (1) were also performed using HBM data obtained from the literature and from documents produced by the Risk Assessment Committee of the European Chemicals Agency (Table 1). For exposure values below the limit of detection (LOD), LOD/2 was used. The Student’s *t*-test was used to compare SRQ values obtained through air and HBM measurements. All data was compiled and analysed using MS Excel 2016.

An RQ or an SRQ < 1 indicates low concern after workers’ exposure to the single substance or the mixture, respectively. Conversely, an RQ or an SRQ > 1 indicates that the tolerable risk level was exceeded, and that exposure is likely to have an impact on workers’ health [31,32].

## 3. Results

### 3.1. Characteristics of the Studies

**Figure 2:**
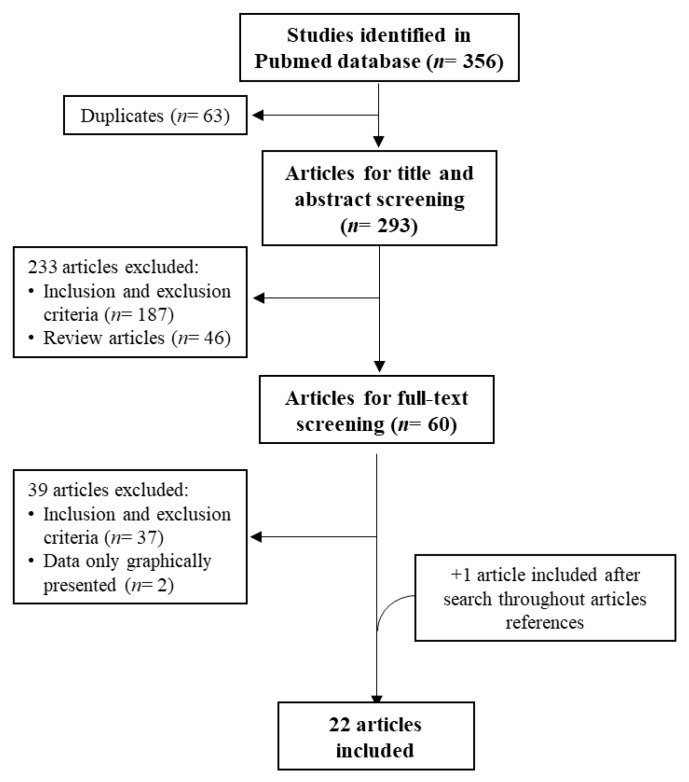
Flowchart of the selection process.

Overall, 356 articles were retrieved from the PubMed search. After removing duplicates, applying selection criteria, and conducting a second search throughout the chosen articles’ references, a total of 22 articles were selected. A flowchart of this selection process is depicted in Figure 2.

Of the 22 articles included in this study, 7 were published before 2010, and only 5 were published in the last five years. Biomarkers of exposure in urine were presented in 19 out of 22 articles, while 5 articles referred biomarkers in blood (blood, plasma and/or erythrocytes) [44,45,46,47,48], 5 articles included biomarkers in exhaled breath condensate (EBC) [49,50,51,52,53], and only 1 article presented measurements in hair and saliva [46]. Besides biomarkers of exposure to Cr, Ni and/or PAHs, most studies also assessed biomarkers of exposure to other substances, such as other heavy metals (Mn, Fe, Al, Hg, As, Be, Pb, Cu, Cd, Co, V), polychlorinated biphenyls (PCBs), and organochlorinated compounds [46,47,48,49,50,51,52,54,55,56,57,58,59,60,61,62]. Table 2 presents urinary levels (median or mean values) retrieved from the selected studies, as well as the description of the respective settings and exposure scenarios. Welding activities were the most frequently assessed, including 9 studies [16,17,44,47,50,52,53,54,55], followed by waste incineration assessed in 4 studies in the context of waste management operations [57,59,60,61].

### 3.2. Overall Exposure Biomarkers in Urine

Most studies with data on exposure biomarkers in urine reported levels of both urinary chromium (U-Cr) and Ni (U-Ni) (*n* = 15) [16,17,44,45,46,47,50,52,53,54,55,56,61,62,63], as displayed in Table 2.

U-Cr was measured in 17 studies. Five studies reported exposure values exceeding the limits previous calculated (Table 2), among workers performing incineration operations, welding, namely flux cored arc welding, stainless steel grinding and electroplating. The highest value was measured among welders at the end of the workday (3.96 µg/g creatinine) [44]. The lowest exposure value (0.14 µg/g creatinine) was measured among dental technicians who prepared prostheses and metal constructions for dental crowns at a dental laboratory [62]. A low exposure level was also observed among harbour workers who worked on lighters and suction dredgers (0.22 µg/g creatinine) [56].

Similarly, U-Ni was determined in 19 studies. Limit values were exceeded in 8 studies (Table 2), among industry workers performing incineration operations, flux cored arc welding, stainless steel grinding, and electroplating, and also among workers performing prosthesis preparation. The highest level of U-Ni measured (12.12 ± 8.31 µg/g creatinine) was observed in workers performing incineration operations and other related activities for more than 3 months and for less than 8 months, in a hazard waste incinerator [61]. The lowest levels of U-Ni levels (0.25 µg/g creatinine) were detected among workers in the production, polishing and shaving of stainless-steel vessels and other metallurgical processes at an iron and steel industry [46].

Levels of PAH metabolites, namely, 1-OHP in urine (U-1-OHP), were assessed in only 3 studies, all conducted with workers performing operations at waste incinerators. None of the exposure levels reported in the studies exceeded the limit values considered in this study (Table 2).

### 3.3. Risk Quotients in Welding Activities with Exposure to Cr(VI) and Ni

Table 3 shows the air levels of Cr(VI) and Ni retrieved from seven studies on welding activities. The exposure levels per type of welding activity, as well as the respective RQ and SRQ, were calculated using Equation (1) (see Section 2) and the tolerable risk levels of substances in air. The SRQ values obtained ranged from 0.12 to 252.8, the latter for settings of Gas Metal Arc Welding with massive or flux cored wire of stainless steel (high exposure group). The SRQ exceeded a value of 1 for ten out of fourteen combinations of Cr and Ni exposure levels (Table 3).

All studies conducted on welding activities also reported U-Cr and U-Ni levels, but none of those considered a combined analysis of the 3 substances (i.e., Cr, Ni, PAHs). Table 4 lists the exposure levels in urine per type of welding activity, as well as the respective RQ and SRQ calculated using Equation (1) and limit values presented in Table 1. An SRQ > 1 was obtained in fourteen out of sixteen combinations of U-Cr and U-Ni exposure levels. In ten combinations, the SRQ exceeded a value of one but the respective U-Cr and U-Ni RQ were below the limit values (RQ < 1). The highest SRQ was estimated among “high exposure” workers, who performed gas metal arc welding with massive or flux cored wire of stainless steel (SRQ = 10.9). In another study, the second highest SRQ was also obtained for workers performing flux cored arc welding (SRQ = 4.43) (Table 4).

**Figure 3:**
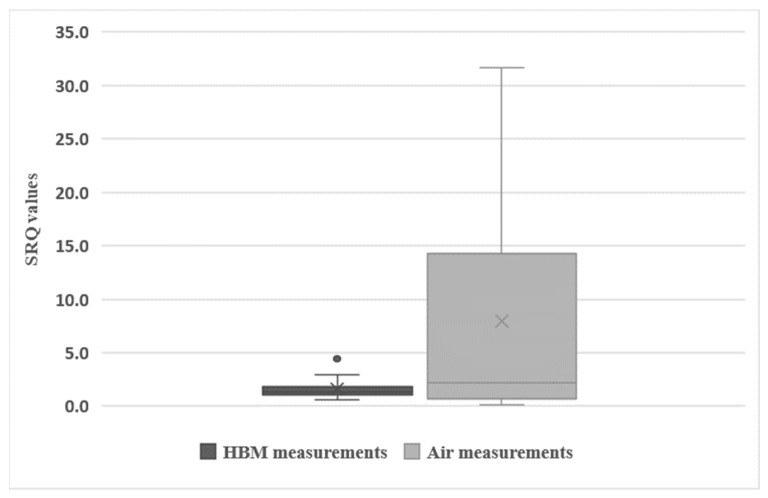
Boxplot showing the SRQ determined using values from air measurements (light grey) or using HBM measurements (dark grey). •—outlier; ×—mean SRQ values (mean (HBM) = 1.62; mean (air) = 7.93).

When comparing the results displayed in Table 3 and Table 4 for the same studies, the SRQ obtained using air measurements were significantly higher (about 5-fold higher) than the SRQ determined using HBM data (*p* = 0.030, Student’s *t*-test). The distribution of SRQ from air measurements and HBM data (leaving out the high exposure group) is displayed in Figure 3. A much higher variability across studies was observed when considering the SRQ values obtained from air measurements.

Among the ten combinations that generated SRQ > 1 based on air Cr and Ni levels, only eight produced SRQ > 1 based on HBM data [17,44,47,52,53,55]. Inversely, in four combinations, SRQ < 1 were derived from external exposure data for gas metal arc welders, shielded metal arc welders, tungsten inert gas welders [16] and flux-cored arc welders of mild steel [47], whereas the corresponding HBM-based SRQ values were 1.74, 1.02, 1.09 and 1.21, respectively, i.e., exceeding the threshold levels of concern.

From another perspective, a more than 17-fold increase in the Cr RQ over Ni RQ was found for the “high exposure group” among gas metal arc welders with massive or flux cored wire of stainless steel [47], suggesting that Cr will be the driver of the mixture toxicity. In line with this observation, a 4-fold increase in Cr RQ over Ni RQ was also noted in another study involving gas metal arc welders [17]. Interestingly, using HBM data, the comparison between the RQ values calculated based on the U-Cr and U-Ni levels showed a 1.8-fold decrease and a 3-fold increase for the “high exposure group” among gas metal arc welders with massive or flux cored wire of stainless steel [47] and for the gas metal arc welders [17], respectively.

### 3.4. Risk Quotients after Exposure to Cr(VI), Ni and PAHs

Only two studies conducted in hazard waste incinerator settings reported biomarkers of exposure in urine for Cr, Ni, and PAHs, but no air measurements were conducted in these studies [57,59] (Table 2). The levels of urinary Cr, Ni and 1-OHP retrieved and the respective RQ and SRQ values are presented in Table 5. For all exposure scenarios described, SRQ were above one, even among workers performing activities considered of low or non-exposure, such as laboratory and administrative activities, respectively. In both studies, workers were exposed to Cr and PAHs (measured through 1-OHP) at levels below the limit values (RQ < 1); nevertheless, values of SRQ above one were obtained for all activities performed (Table 5).

### 3.5. Effect Biomarkers

Effect biomarkers were analysed in 6 out of the 22 articles included (Table 6). Three studies assessed chromosomal damage, using the micronucleus assay [56,58,61], and two of them also assessed sister chromatid exchanges (SCE) [56,58]; one study analysed DNA damage by the comet assay [61]. No significant increases in micronuclei and SCE frequencies, or in DNA strand break levels were observed among exposed groups when compared to the control groups. Two studies on welders analysed biomarkers of oxidative stress in EBC [50,53]. A significant increase in H_2_O_2_/tyrosine, nitrate/tyrosine and cotinine concentrations was shown in welders’ EBC compared to controls [50]. However, in tungsten inert gas welders, the biomarkers analysed in EBC only differed significantly between workers’ shifts [53]. Another study on welders also measured oxidatively modified guanosines, but no differences were observed across the different welding techniques after creatinine adjustment. Moreover, no comparison was made between exposed and control groups in that study [47].

## 4. Discussion

The identification of all chemicals present in an occupational setting and the fluctuations in exposures levels during the wide variety of tasks performed in a workday or week can be quite challenging [64]. The knowledge regarding co-exposure to chemicals is quite limited for many workplaces, and the approach commonly followed to identify risk factors is often focused on single chemicals directly resulting from the process or that are used as raw materials.

HBM is an important tool to identify and quantify workers’ exposure to emerging or well-known contaminants and can further assist in the evaluation and demonstration of the effectiveness of policy actions or measures already taken at a company level to reduce exposure [39]. In this work, we observed that recent studies conducted in European Union countries and reporting biomarkers of exposure to Cr(VI), Ni and PAHs are scarce. These findings highlight that in most occupational studies and in industrial hygiene routines, the measurement of external exposure remains the common practice to assess workers exposure. However, HBM data is of added value, particularly in the case of substances for which exposure by ingestion, due to hand-to-mouth contact (the case of metals), or by dermal absorption (the case of PAHs) is relevant, as it reflects exposure from all sources and routes of exposure. In this study, we conducted a mixture risk assessment exercise based on both external and internal exposure levels of Cr(VI), Ni and PAHs reported in the literature.

A first general observation was that the SRQ values were significantly higher when using air measurements than using HBM data (urinary biomarkers). Additionally, a higher variability among SRQ values obtained from air measurements was observed, compared with the SRQ values calculated from HBM measurements. The air concentrations of chemicals are influenced by many external factors such as temperature, ventilation, the type of activities being performed, among others, while they do not account, for example, for the effect of individual protection equipment. The lower RQ and SRQ values obtained using HBM data and the lower associated variability, may be mainly related to the use of respiratory protection equipment by workers that will reduce exposure. In addition, uncertainties related to the air–urinary levels correlations, particularly at the low exposure levels, may contribute to this observation. Considering that HBM results give a more accurate estimate of the actual exposure level because they reflect the internal doses of chemicals but also that external exposure levels are usually assessed under occupational health surveillance programs, it seems that this latter approach is able to adequately detect health risks from exposure to single chemicals. Nevertheless, it is important to note that HBM data, while covering all possible routes of workers’ exposure, accounting for individual variability (e.g., in chemicals metabolism) and, hence, reflecting the internal doses of chemicals, also provide a more reliable exposure assessment to perform MRA.

Focusing on welding activities where co-exposure to Cr and Ni occurs, an HBM-based SRQ revealed exposure levels of concern in most of the activities, with several exceedances of the limit values, even in the most recent studies. These observations raise concern, since Cr(VI) and Ni are potent human carcinogens [3,4], and cancer risks from welding activities, in particular stainless-steel welding, have been a matter of concern [65]. In some of the analysed studies, welding activities generated RQ < 1 based on U-Cr or U-Ni; however, the associated SRQ was above one. Cr and Ni share genotoxic and non-genotoxic MoAs and both substances may induce a similar health outcome (lung cancer). However, interactive effects are not often considered when performing workplace risk assessment, even though the EU Chemical Agents Directive (98/24/EC) clearly states that in the case of activities involving exposure to several hazardous chemical agents, the risk shall be assessed on the basis of the risk presented by all such chemical agents in combination. Indeed, in the occupational health field, combined effects are never taken into account in individual Observed Effect Levels (OELs) although the employer should assess the risks related to the combined effects of chemicals in the work environment by, e.g., using the hazard index (HI) approach. For example, the EU CAD directive states that “in the case of activities involving exposure to several hazardous chemical agents, the risk shall be assessed on the basis of the risk presented by all such chemical agents in combination”. In some countries, such as Finland, the employers have been instructed and advised that they should take possible combined effects into account if the substances have the same MoA. Guidance on HI approach is provided, but unfortunately, this is generally applied mostly in the case of irritant solvents, not in the case of carcinogens. This seems to be the general situation in many European countries, i.e., even though legislation requires consideration of combined effects, this is not very effectively performed in practice. This means that the potential impact of industrial chemicals on workers’ health might be overlooked. In light of these findings, regulatory actions put in place at these occupational environments should be re-evaluated in order to reduce workers’ exposure.

Although in many of the analysed studies, urinary levels of Cr and Ni were assessed, only two studies focused on hazardous waste incineration reported HBM data for Cr, Ni and PAHs. However, the co-occurrence of these substances has been recognized in many other industrial activities [66], e.g., in electromechanical repair and car painting centres, sewing work rooms, chemical analysis laboratories [67], coke oven plants, aluminium production, iron and steel founding and welding industries, among others [18,19,20], showing the paucity of data allowing the assessment of the risks related to those mixtures exposure.

In the context of activities conducted at incinerators, RQ < 1 values were obtained based on U-Cr or U-1-OHP levels that resulted in SRQ > 1. Surprisingly, these results were also observed among administrative workers from the incinerator industries. These findings indicate that if only exposure to single metals is considered, no concern will be raised, reinforcing the importance of considering the mixture for a realistic risk assessment. Furthermore, our results regarding administrative staff highlight the fact that these workers from the same industrial facilities can also present a certain level of exposure possibly due to not wearing protection equipment. Those workers should also be aware of the risks they incur in and should be enrolled in occupational health surveillance schemes or in other occupational health interventions (e.g., dedicated health surveillance programs).

Exposure to chemical mixtures, namely, substances exhibiting similar MoAs, may lead to adverse health effects, even at exposures below the chemical’s threshold level. Moreover, from the exposure to multiple chemicals, toxicokinetic and/or toxicodynamic interactions may occur, resulting in new toxic effects usually not observed for single exposures, such as synergism [30,64]. An in vitro study showed that human lung fibroblasts exposed to Cr(VI) and PAHs metabolites resulted in synergistic mutagenic and cell transformation effects [23]. However, an in vivo study demonstrated tissue-specific effects in the liver and gastrointestinal tract, namely, at molecular, gene expression and histopathological level, due to exposure to environmentally relevant Cr(VI) and BaP doses. For instance, BaP reduced mice body weight by more than 10%, but this effect was not modified by co-exposure to Cr(VI). Co-exposure to BaP and Cr(VI) also induced an additive effect in crypt hyperplasia in the small intestine of mice, while in the liver, both substances cause more severe histopathological lesions than those expected from the sum of each single compound (more than additive). Nevertheless, regarding genotoxic effects, co-exposure increased the phosphor-cH2AX-positive cells observed for each single substance, but not in an additive way [25]. These observations in experimental systems have been strengthen by the findings of epidemiological studies contributing to a higher weight of evidence. A study conducted by Wang et al. (2015) on coke oven workers in China detected urinary concentrations of heavy metals, including Cr and Ni, and PAHs metabolites in most of the samples, and reported significantly higher oxidative stress biomarkers among workers exposed to high levels of PAHs and metals, compared to those only exposed to one substance [20]. A study by Campo et al. (2020) observed an additive effect between metals and PAHs on 8-oxodG levels in electric steel foundry workers in Tunisia [68]. Moreover, in a recent study by Zeng et al. (2022), a positive association between co-exposure to PAHs and metals and oxidative stress (dose–response curve of 3-OHPhe or Al with 8-iso-PGF2α) measured among coke oven works was suggestive of a synergistic effect [69].

Effect biomarkers allow us to identify early biological effects from chemicals exposure and contribute to establish exposure–response relationships, explore mechanisms that underlie health outcomes and increase the knowledge on the biological plausibility of epidemiological associations between exposure and adverse health effects [70]. However, in epidemiological studies, effect biomarkers have been used to a lesser extent than exposure biomarkers, thus constituting an understudied field [64]. Among the studies analysed, only six reported data on effect biomarkers [47,50,53,56,58,61]. The most used biomarkers targeted genetic damage: chromosome alterations (MN and SCE in blood lymphocytes), DNA damage (comet assay in blood leukocytes), oxidative DNA adducts formation (8-oxodGuo) or oxidative stress. Comparisons of the values obtained for those biomarkers between exposed and control groups or among workers groups with different exposure levels did not reveal any significant increase associated with exposure, irrespective of the effect biomarker used. Two studies reported statistical comparisons of MN frequencies between incineration workers or harbour dredgers and lighters exposed to Cr and Ni and the respective control groups [56,61]. Both studies failed to find increased MN frequencies in the exposed group. These findings contrast with those from other studies on occupational exposure to Cr(VI) or to Cr(VI) and Ni that reported significantly higher genetic damage in workers comparatively to controls [71,72,73]. Such differences might also be due to timing issues during samples collection, e.g., the time elapsing between exposure and samples collection, which is determinant to detect DNA damage in blood cells and to other factors related to the study design, e.g., a too small number of participants. More occupational studies comprising, preferably, exposure and effect biomarkers are needed to provide more evidence on their value to link exposure and health effects.

Some limitations in this study must be acknowledged. A considerable degree of heterogeneity was observed amongst studies. Data was stratified by workday, pre- or post-shift sampling, and by duration of exposure, such as hours or months. In terms of statistical treatment of data, some studies presented data per individual measurements; others presented arithmetic means, geometric means, or medians. The units used to express urinary biomarkers also varied from adjustment to µg/g of creatinine, to µg/L, µmol/mol of creatinine, and nmol/L. These aspects possibly prevented us from making a more accurate comparison of the data retrieved. In the HBM4EU initiative, the importance of harmonizing methodologies and designs in HBM studies has been highlighted in recent publications [40,74], reinforcing the importance of joint efforts to produce comparable data that can be further used to regulatory actions in the context of different frameworks. Contextual information was also lacking in some studies, such as information on possible exposure routes. However, since we analysed HBM data, all exposure routes were considered in our RQ and SRQ calculations. In the analysed studies, there were also exposure biomarkers in blood cells, plasma or exhaled breath condensate, although we only considered urinary exposure biomarkers. However, these are the most commonly used in HBM studies due to its simplicity of sampling, non-invasive nature and the existing validated methods for measurement.

Another important limitation were the uncertainties related to the conversion of urinary levels from air levels to be used as limit values for our MRA. For Cr, we have used a regression equation that moderately correlates new exposure data from welders [41]; for Ni, regression equations are also very unsure, especially at low exposure levels and in the case of poorly soluble Ni compounds present in welding [37]; for 1-OHP, some uncertainties must be also considered due to the fact that for PAHs we are often dealing with mixtures with variable pyrene vs. BaP ratios. Furthermore, regarding the attempt to identify the potential driver of the mixtures’ toxicity, a simplified approach, i.e., the comparison of the RQ values calculated for each single component, was used. It is important to note that the toxicity of each component in a mixture can be substantially different, and that the effect of the mixture thereby depends not only on each chemical’s concentration but also on its relative contribution to the toxicity observed. This means that a small quantity of a very potent carcinogen (e.g., benzo[a]pyrene) in the mixture can have a much larger biological effect than a large amount of a less potent chemical (e.g., Ni). Nevertheless, the RQ value accounts for the toxicity of the chemical because it allows the comparison of exposure doses or concentrations with available reference values (e.g., no observed effect or lowest effect concentrations) derived from toxicity data.

A recent survey on field experts on the use of HBM data in chemical risk assessment revealed that the lack of HBM-based guidance values for the majority of substances has greatly limited the use of HBM data for risk assessment [75]. There is no agreement on the acceptable risk levels in Europe for occupational activities involving carcinogenic substances. Given that this was a crucial point for this work, because this can significantly impact the RQ and SRQ results, the topic was debated with an expert group in occupational health and risk assessment. Those experts recognized that the tolerable risk level of 1:4000, as defined in the German Technical Rules for Hazardous Substances (TRGS), produced by the Committee on Hazardous Substances (AGS) of the Federal Institute for Occupational Safety and Health), that describe the state-of-the-art knowledge on occupational medicine and industrial hygiene for activities with hazardous substances, could be followed. According to TRGS, above the defined cross-substance tolerable risk limit of 4:1000, there is a high risk for activities involving carcinogenic substances that is classified as not tolerable. Likewise, the “Risk Concept for Carcinogenic Substances” of the AGS offers a benchmark for comparison and assessment of exposure to carcinogenic substances, including limit values (in air) for the three components of the mixture under study, which was considered an advantage, assuming that those values’ derivation followed a similar rationale, decreasing the possible uncertainty of selecting limit values derived by different approaches or for different population groups.

A linear no-threshold dose–response relationship has been generally assumed for carcinogenic chemicals, regarding cancer risk assessment. This relationship applies to Cr(VI) that acts as a direct genotoxic carcinogen (Cr-ternary DNA adduct is considered the critical lesion) for which no threshold can be assumed and linear extrapolation is commonly applied [36]. Likewise, many PAHs, including BaP, are carcinogenic and share the same direct genotoxic mechanism of action (metabolic activation to electrophilic intermediates and formation of DNA adducts leading to mutation), indicating a non-threshold dose-effect relationship [38]. However, nickel compounds act through indirect and secondary (via inflammation) genotoxicity mechanisms, and a threshold MoA for their carcinogenic effects [37] is considered. For these compounds, a “hockey stick”-shaped dose–response curve has been assumed, which likely contains a breakpoint at 0.005 mg/m^3^ or 0.006 mg/m^3^ [37]. Given that Ni does not produce effects at exposures below the indicated limit value in air, only the effect of Cr(VI) (exposure to the binary mixture) or of the mixture of PAHs and Cr(VI) (ternary mixture) should be considered at such exposure levels. This does not affect the overall RQ and SRQ values calculated from air measurements data (Table 3), in which a single study described air values below the limit value but contributing to a SRQ above 1 [53].

Several authors have addressed some potential limitations of the CA mathematical model that may hamper its application to environmentally realistic assessment cases, where chemicals with different modes of action or showing different dose–response relationships can co-occur. This may be the case of mixtures of genotoxicants, for which the application of the CA model is not consensual. Kortemkamp et al. (2014) [76] assessed the applicability of either CA or IA to several mixtures of aneugenic and clastogenic chemicals by using the micronucleus assay in CHO-K1 cells. The authors concluded that grouping the chemicals into common assessment groups and using hybrid CA/IA prediction models could provide a better prediction for the effect of mixtures consisting of chemicals with different mechanisms of genotoxic action than using either the CA or the IA model. In such cases, the application of CA did not offer a good description of the observed effect and neither did the IA that led to an underestimation of the joint effect. The uncertainties associated with estimating low-level effects also precluded firm conclusions regarding the use of the IA at such low levels. In addition, it is worth noting that the use of the IA requires knowledge on the precise effect magnitude that each component would provoke if present individually at the concentration found in the mixture. This information is only accessible through comprehensive dose–response analysis of each mixture component, which is difficult to find [30]. With this perspective, the use of the CA model to the mixture herein assessed under realistic exposure scenarios seemed to be the best option, even though Ni exposure will probably not contribute to the joint effect at exposure levels below the dose–response breakpoint.

As the substances in the mixture under study are carcinogenic to humans, it is recommended that exposure should be reduced to values close to zero among workers exposed to these substances. It is also important to keep in mind that within occupational settings, substitution of such carcinogens by other substances may not be possible in a short timeframe, and therefore, occupational exposure to non-threshold carcinogens should be limited to levels as low as reasonably achievable (ALARA principle) to protect workers’ health.

Overall, this study highlights that risk assessment frameworks only focused on single substances are insufficient in light of the increasing evidence underlining the importance of considering cumulative exposure to multiple chemicals in order to more adequately estimate the risks they pose [27]. Understanding exposure to realistic concentrations of chemical mixtures and their associated health effects will require increased collaboration across different scientific fields, namely, toxicology, epidemiology, exposure science, risk assessment and statistics, for a proper integration of data from each discipline [26]. More MRA studies based on HBM data obtained in real occupational exposure scenarios are needed for other industrial settings. Aiming to contribute to a reliable assessment and management of chemical risks, the HBM4EU initiative is currently building capacities to establish a European Human Biomonitoring Platform in order to harmonize HBM activities in EU countries. This will deliver comparable data on human exposure to chemicals and mixtures of chemicals as a basis for policy making to improve chemical safety (https://www.hbm4eu.eu/about-hbm4eu/, accessed on 10 March 2022). An occupational study aiming to assess external and internal exposure, as well as effect biomarkers, in workers from an aircraft maintenance company exposed to Cr(VI), Ni and PAHs is already ongoing. The results are expected to give a better insight into the co-exposure and early health effects of this mixture in exposed workers.

## Figures and Tables

**Table 1:** Tolerable risk levels and limit values used for risk assessment calculations.

Substances	Tolerable Levels in Air (µg/m^3^) ^a^	Limit Values in Urine	
		µg/g Creatinine	µg/L
**Chromium (VI)**	1.0	1.20 ^c^	1.63 ^d^
**Nickel ^b^**	6.0	2.21 ^d^	3.00 ^e^
**Benzo(a)pyrene**	0.07	1.03 ^d^ (for 1-OHP)	1.40 ^f^ (for 1-OHP)

^a^ Limit values established in the Technical Rules for Hazardous Substances, AGS, 2021 [43]; ^b^ Nickel metal, nickel oxide, nickel carbonate, nickel sulphide, sulfidic ores [43]; ^c^ Based on the regression analysis conducted by Viegas et al. (2022) on U-Cr and inhalable Cr(VI) levels found in welders; equation: y = 0.647 + 0.541x [41]; ^d^ Conversion from µg/g creatinine to µg/L and from µg/L to µg/g creatinine using the standard creatinine value (~1.36 g/L urine); ^e^ Value recommended by the SCOEL for a biological guidance value (BGV) for nickel, as referred in [37]; ^f^ Calculated based on the dose–response relationship between BaP concentration in air and 1-OHP in urine; RAC note from ECHA, 2018 [38].

**Table 2:** Occupational settings, exposure scenarios and median (±SD) exposure values for exposure biomarkers in urine reported in the selected studies (*n* = 19).

Occupational Setting	Activities	Biomarkers of Exposure in Urine (µg/g Creatinine)			Ref.
		U-Cr	U-Ni	U-1-OHP	
	Incinerator operations, boiler and furnace maintenance, control panel, and waste-gas-washing	0.39 ± 0.24 ^a^	3.7 ± 1.9 ^a^	<0.04 ± 0.3 ^a^	[57]
		0.26 ^a^	3.03 ^a^	0.1 ^a^	[59]
Waste incineration			4.8 ± 3.2 (<4; 11.0) ^a^	0.20 ^a^	[60]
		0.50 ± 0.58 (exposure 1–3 months) 1.89 ± 4.59 (exposure 3–8 months) 0.26 ± 0.24 ^a^ (exposure 8–11 months)	9.18 ± 7.47 (exposure 1–3 months) 12.12 ± 8.31 (exposure 3–8 months) 7.69 ± 5.57 ^a^ (exposure 8–11 months)		[61]
Manufacture of railway vehicles	Welding ^e^	0.72 (IQR 0.58–1.20)	1.56 (IQR 1.01–2.48)		[52]
Welding industries		0.32 (IQR 0.97) (pre-shift) 0.54 (IQR 1.39) (post-shift)	1.11 (IQR 1.6) (pre-shift) 1.47 (IQR 2.27) (post-shift)		[50]
		2.3 (0.7–7.3) ^b^	1.5 (0.5–3.2) ^b^		[55]
	Flux cored arc welding	3.8 (0.48–18.0) (first void) 3.2 (<0.24–30.1) (before work) 3.96 (0.34–40.7) (after work) ^a^	1.9 (1.05–5.59) (first void) 2.7 (1.11–4.37) (before work) 2.5 (0.56–5.0) (after work) ^a^		[44]
	Flux cored and gas metal arc welding	<1.35 (IQR <0.74–<3.24)	<2.56 (IQR <1.37–<4.39)		[17]
	Gas metal arc welding	0.39	1.57		[54]
	Tungsten inert gas welding	0.71 (0.36–1.27) (before Friday shift) 0.74 (0.41–1.21) (after Friday shift) 0.59 (0.26–1.00) (after weekend break)	0.76 (0.37–1.40) (before Friday shift) 1.11 (0.59–0.79) (after Friday shift) 0.83 (0.31–1.38) (after weekend break)		[53]
Shipyards and other industries		0.85 (IQR 0.53–1.32) (pre-shift) 0.90 (IQR 0.56–1.55) (post-shift)	1.53 (IQR 1.05–3.37) (pre-shift) 1.67 (IQR 0.89–2.97) (post-shift)		[16]
	Gas metal arc welders with massive or flux cored wire of stainless steel; Flux-cored arc welding of mild steel; Tungsten inert gas welding	0.88 ^d^	2.07 ^d^		[47]
	Stainless steel grinding	1.6 (<0.15–4.6) (first void) 1.40 (<0.14–4.5) (before work) 1.40 (<0.13–5.5) (post shift)	3.79 (0.68–10.6) (first void) 3.39 (0.25–11.1) (before work) 4.56 (<0.53–11.5) (post shift)		[45]
Harbour	Harbour dredgers and lighters	0.22 ^d^	0.7 (0.1–7.3)		[56]
Iron and steel industry	Production, polishing and shaving of stainless-steel vessels and other metallurgical processes	0.42 (0.10–19.65)	0.25 (0.12–51.01)		[46]
Dental laboratory	Preparation of prostheses and of metal constructions for dental crowns	0.14 ^c,d^	4.35 ^c,d^		[62]
Electroplating companies	Processes of electroplating, preparatory work, maintenance activities, polishing of electroplated items, and ancillary tasks	1.10 ^d^ 1.47 (electroplating workers) ^d^	4.26 ^d^ 4.88 (electroplating workers) ^d^		[63]
Workshops specializing in the processing of beryls	Gemstone cutting		≤4 h/week: 1.18 (pre-shift); 0.85 (post-shift) ^d^ >4 h/week: 0.81 (pre-shift); 0.66 (post-shift) ^d^		[58]

U-Cr, Urinary chromium; U-Ni, Urinary nickel; U-1-OHP, Urinary 1-hydroxypyrene; IQR, Interquartile Range; ^a^ Mean value; ^b^ Geometric means; ^c^ Calculated arithmetic mean of pooled urine samples from exposed workers; ^d^ Reported exposure values converted to µg/g creatinine; ^e^ Unspecified welding activity.

**Table 3:** External exposure levels of Cr and Ni (air measurements) of workers performing welding activities, and values obtained for RQ and SRQ.

Welding Activity	Cr		Ni		SRQ	Ref.
	Exposure Level (µg/m^3^) ^a^	RQ	Exposure Level (µg/m^3^)	RQ		
Welding ^f^	20.0 ^b,c,e^	**20.0**	70.0 ^c,e^	**11.7**	**31.7**	[52]
	6.30 ^b,g,h^	**6.30**	2.80 ^g,h^	0.47	**6.77**	[55]
Flux cored arc welding	11.3 ^i^	**11.3**	50.4 ^a,i^	**8.40**	**19.7**	[44]
	1.80 ^b,c,d^	**1.80**	2.15 ^c,d^	0.36	**2.16**	[17]
Gas metal arc welding	7.80 ^b,c^	**7.80**	11.0 ^c^	**1.83**	**9.63**	[17]
	0.24 ^h^	0.24	2.70 ^h^	0.45	0.69	[16]
Shielded metal arc welding	0.04 ^h^	0.04	0.49 ^h^	0.082	0.12	[16]
	17.0 ^b,c^	**17.0**	4.00 ^c^	0.67	**17.7**	[17]
Tungsten inert gas welding	10.5 ^b,c^	**10.5**	2.55 ^c,d^	0.43	**10.9**	[17]
	0.23 ^h^	0.23	0.67 ^h^	0.11	0.34	[16]
	1.00 ^d,i^	1.00	1.00 ^i^	0.17	**1.17**	[53]
	1.35 ^b,d,i^	**1.35**	0.75 ^d,i^	0.13	**1.48**	[47]
Gas metal arc welding with massive or flux cored wire of stainless steel (high exposure group)	239.0 ^b,i^	**239.0**	82.5 ^i^	**13.8**	**252.8**	[47]
Flux-cored arc welding of mild steel	0.60 ^b,d,i^	0.60	0.85 ^d,i^	0.14	0.74	[47]

Tolerable levels: 1 µg/m^3^ of Cr(VI) compounds and 6 µg/m^3^ of nickel compounds [43]; ^a^ Expressed as median level, except in reference [44] that presents the mean value; ^b^ Data presented for chromium, without specifying its ionic form. It was assumed that authors were referring to Cr(VI); ^c^ Inhalable fraction; ^d^ Values < LOD; LOD/2 was determined; ^e^ No mean or median values presented; the highest value was chosen; ^f^ Unspecified welding activity; ^g^ Geometric mean; ^h^ Respirable fraction; ^i^ Unspecified fraction considered as inhalable fraction; RQ > 1 and SRQ > 1 in bold.

**Table 4:** Exposure levels of Cr and Ni in urine (HBM) of workers performing welding activities, and values obtained for RQ and SRQ.

Welding Activity	U-Cr		U-Ni		SRQ	Ref.
	Exposure Level	RQ	Exposure Level	RQ		
Welding ^d^	0.72 µg/g creat	0.60	1.56 µg/g creat ^e^	0.71	**1.31**	[52]
	2.30 µg/g creat ^b^	**1.92**	3.10 µg/L ^b^	**1.03**	**2.95**	[55]
	0.54 µg/g creat (post-shift)	0.45	1.47 µg/g creat ^e^ (post-shift)	0.67	**1.12**	[50]
Flux cored arc welding	3.96 µg/g creat (after work) ^a^	**3.30**	2.50 µg/g creat (after work) ^a,e^	**1.13**	**4.43**	[44]
	0.43 µg/g creat ^c^	0.36	2.83 µg/L	0.94	**1.30**	[17]
Gas metal arc welding	0.83 µg/g creat ^c^	0.69	3.76 µg/L ^c^	**1.25**	**1.94**	[17]
	0.39 µg/g creat	0.33	1.57 µg/g creat ^e^	0.71	**1.04**	[54]
	0.94 µg/g creat (post-shift)	0.78	2.87 µg/L (post-shift)	0.96	**1.74**	[16]
Shielded metal arc welding	0.74 µg/g creat (post-shift)	0.62	1.20 µg/L (post-shift)	0.40	**1.02**	[16]
	1.17 µg/g creat ^c^	0.98	1.98 µg/L	0.66	**1.64**	[17]
Tungsten inert gas welding	0.61 µg/g creat ^c^	0.51	0.75 µg/L ^c^	0.25	0.76	[17]
	0.78 µg/g creat (post-shift)	0.65	1.31 µg/L (post-shift)	0.44	**1.09**	[16]
	0.74 µg/g creat (after Friday shift)	0.62	1.11 µg/g creat ^e^ (after Friday shift)	0.50	**1.12**	[53]
	0.50 µg/L ^c,e^	0.31	0.75 µg/L ^c^	0.25	0.56	[47]
Gas metal arc welders with massive or flux cored wire of stainless steel (high exposure group)	13.53 µg/L ^e^	**8.29**	7.92 µg/L	**2.64**	**10.9**	[47]
Flux-cored arc welding of mild steel	0.50 µg/L ^c^	0.31	2.70 µg/L	0.90	**1.21**	[47]

Limit values: 1.2 µg/g creatinine for total chromium [41]; 3 µg/L for nickel [37]; U-Cr, Urinary chromium; U-Ni, Urinary nickel; ^a^ Mean value; ^b^ Geometric means; ^c^ Values <LOD; LOD/2 was determined; ^d^ Unspecified welding activity; ^e^ Interconversion from µg/g creatinine to µg/L of urine using the standard creatinine value (~1.36 g/L urine), for RQ calculations; RQ > 1 and SRQ > 1 in bold.

**Table 5:** Occupational exposure to Cr, Ni and PAHs in hazard waste incinerators, based on biomarkers of exposure in urine, and respective RQ and SRQ.

Activities	U-Cr		U-Ni		U-1-OHP		SRQ	Ref.
	Exposure Level (µg/g Creat)	RQ	Exposure Level (µg/g Creat)	RQ	Exposure Level (µg/g Creat)	RQ		
Incinerator operations, boiler and furnace maintenance, control panel, and waste-gas-washing	0.39	0.33	3.70 ^b^	**1.68**	0.30 ^b^	0.29	**2.30**	[57]
	0.26	0.22	3.03 ^b^	**1.37**	0.10 ^b^	0.10	**1.69**	[59]
Laboratory activities	0.57	0.48	2.30 ^b^	**1.04**	0.20 ^b^	0.19	**1.71**	[57]
	0.19	0.16	2.55 ^b^	**1.16**	0.02 ^a,b^	0.019	**1.34**	[59]
Administrative activities	0.22	0.18	3.60 ^b^	**1.63**	0.02 ^a,b^	0.019	**1.83**	[57]
	0.26	0.22	2.34 ^b^	**1.06**	0.02 ^a,b^	0.019	**1.30**	[59]

Limit values: 1.2 µg/g creatinine for total chromium [41]; 3 µg/L for nickel [37]; 1.4 µg/L for 1-OHP [38]; ^a^ Values < LOD (<0.04 µg/g creat); LOD/2 was determined; ^b^ Interconversion from µg/g creatinine to µg/L of urine using the standard creatinine value (~1.36 g/L urine), for RQ calculations; RQ > 1 and SRQ > 1 in bold.

**Table 6:** Reported outcomes observed in biomarkers of effect.

Activities [Ref.]	Exposure Biomarkers	Effect Biomarkers	Overall Outcomes		SRQ
Harbour dredgers and lighters [56]	U-Cr (0.22 µg/g creat) and U-Ni (0.7 µg/g creat)	MN	Significantly higher frequency in controls vs. exposed	Controls: 14.2 MNs/1000 cells; Exposed: 11.6 MNs/1000 cells	0.36
		SCE	No significant differences between exposed and controls	Controls: 10.2 SCE rate; Exposed: 10.0 SCE rate	
Incinerator operations, boiler and furnace maintenance, control panel, and waste-gas-washing [61]	U-Cr (short-0.5 ± 0.58; medium-1.89 ± 4.59; long-exposure-0.26 ± 0.24 µg/g creat) U-Ni (short-9.18 ± 7.47; medium- 12.12 ± 8.31; long-exposure-7.69 ± 5.7 µg/g creat)	MN	No significant differences between exposed and controls	Controls: 1.3/1000 cells Short-exposure group: 1.6/1000 cells, Medium-exposure group: 1.7/1000 cells Long-exposure group: 1.7/1000 cells	**2.10 (long-exposure)**
		Comet assay		Short exposure group: 6.5 tail factor; Medium exposure group: 6.3 tail factor; Long exposure group: 6.7 tail factor Controls: 7.1 tail factor	
Gemstone cutting [58]	U-Ni ≤4 h/week: 1.18 µg/g creat (pre-shift); 0.85 (post-shift) >4 h/week: 0.81 (pre-shift); 0.66 (post-shift)	SCE	No significant differences between exposed groups	Short exposure group: 9.91 SCE/cell; Long exposure group: 9.70 SCE/ cell	-
Tungsten inert gas welding [53]	U-Cr 0.71 µg/g creat (0.36–1.27) (T1) 0.74 µg/g creat (0.41–1.21) T2) 0.59 µg/g creat (0.26–1.00) (T3) U-Ni 0.76 µg/g creat (0.37–1.40) (T1) 1.11 µg/g creat (0.59–0.79) (T2) 0.83 µg/g creat (0.31–1.38) (T3)	MDA-EBC	Significant decrease at T2	2.79 nM, 2.98 nM and 2.43 nM at T0, T1 and T2, respectively	**1.12**
		HNE-EBC	Significant increase at T0 than at T2	0.53 nM, 0.48 nM and 0.51 nM at T0, T1 and T2, respectively	
		H_2_O_2_-EBC	Significant increase at T1 than at T0 and T2	0.18, 0.25, and 0.16 μM at T0, T1 and T2, respectively	
Welding [50]	U-Cr (0.32 µg/g creat (IQR 0.97) (pre-shift) 0.54 µg/g creat (IQR 1.39) (post-shift) U-Ni 1.11 µg/g creat (IQR 1.6) (pre-shift) 1.47 µg/g creat (IQR 2.27) (post-shift)	H_2_O_2_-EBC	**Significant increase in welders vs. controls**	56 ± 19 pM/µg vs. 6.6 ± 2.2 pM/µg	**1.12**
		Nitrate/tyrosine ratio		0.71 ± 0.13 nM/µg vs. 0.22 ± 0.04 nM/µg	
Gas metal arc welders with massive or flux cored wire of stainless steel; Flux-cored arc welding of mild steel; Tungsten inert gas welding in shipyards industries [47]	U-Cr (0.88 µg/g creat) U-Ni (2.07 µg/g creat)	Urinary 8-oxoGuo	No significant differences between welding techniques (adjusted for creatinine)	High-exposure group: 6.59 µg/g creat; Flux-cored arc welding of mild steel: 7.62 µg/g creat; Tungsten inert gas welders: 6.41 µg/g creat	0.56 for TIGW
		Urinary 8-oxodGuo		High-exposure group: 4.28 µg/g creat; Flux-cored arc welding of mild steel: 3.79 µg/g creat; Tungsten inert gas welders: 4.41 µg/g creat	**10.9** for high exposure group ^a^
		8-oxodGuo/10^6^dGuo in white blood cells		High-exposure group: 5.53/10^6^dGuo; Flux-cored arc welding of mild steel: 4.79/10^6^dGuo; Tungsten inert gas welders: 1.98/10^6^dGuo	**1.21** for FCAW of mild steel

8-oxodG: 8-oxo-7,8-dihydro-2′-deoxyguanosine; 8-oxoGuo: 8-oxo-7,8-dihydroguanosine; 10^6^dGuo: 10^6^ 2′-deoxyguanoside; EBC: exhaled breath condensate; FCAW, flux cored arc welding; HNE: 4-hydroxynonenal; MDA: malondialdehyde; MN: micronuclei; SCE: Sister chromatid exchange; TIGW, tungsten inert gas welding; T0, before Friday shift; T1, after Friday shift; T2, before Monday shift; ^a^ High exposure group corresponding to workers performing gas metal arc welders with massive or flux cored wire of stainless steel.

## Data Availability

Not applicable.

## Supplementary Materials

The following supporting information can be downloaded at: https://www.mdpi.com/article/10.3390/toxics10080431/s1, Table S1: Database searches.
