# Supplementary material for: Occupational Exposure to Hexavalent Chromium, Nickel and PAHs: A Mixtures Risk Assessment Approach Based on Literature Exposure Data from European Countries
Source: Toxics. 2022 Jul 29;10(8):431. doi: 10.3390/toxics10080431 (PMC9414170; doi:10.3390/toxics10080431)
Supplement: Supplementary file 1 [file toxics-10-00431-s001.zip › toxics-1796337-supplementary.pdf]

Table S1: Database searches.

Combinations searched	Search terms (Filters: Humans/Journal Articles/Languages English, Spanish, French or Portuguese, 2000-2020)	Results
PAHs + Ni	("human monitoring" OR "human biological monitoring" OR "occupational monitoring" OR "occupational exposure" OR "biomarkers of exposure" OR worker* OR "human exposure" OR "occupational exposure" OR "occupational exposure" [MeSH Terms] OR "occupational settings" OR "industrial settings" OR "occupational hygiene" OR "industrial hygiene" OR "human biomonitoring" OR "biomonitoring" [MeSH Terms] OR "exposure monitoring" OR "exposure at the workplace" OR "workplace" OR "occupational hazard" OR "occupational health surveillance") AND (("Polycyclic aromatic hydrocarbons" OR "benzo(a)pyrene" OR pyrene* OR PAHs OR PAHs [MeSH Terms]) AND (nickel OR ni))	**50**
PAHs + Cr(VI)	("human monitoring" OR "human biological monitoring" OR "occupational monitoring" OR "occupational exposure" OR "biomarkers of exposure" OR worker* OR "human exposure" OR "occupational exposure" OR "occupational exposure" [MeSH Terms] OR "occupational settings" OR "industrial settings" OR "occupational hygiene" OR "industrial hygiene" OR "human biomonitoring" OR "biomonitoring" [MeSH Terms] OR "exposure monitoring" OR "exposure at the workplace" OR "workplace" OR "occupational hazard" OR "occupational health surveillance") AND (("Polycyclic aromatic hydrocarbons" OR "benzo(a)pyrene" OR pyrene* OR PAHs OR PAHs [MeSH Terms]) AND ("hexavalent chromium" OR "Cr(VI)" OR "chromium" OR "chromate" OR "chromium (VI)")	**35**
Ni + Cr(VI)	("human monitoring" OR "human biological monitoring" OR "occupational monitoring" OR "occupational exposure" OR "biomarkers of exposure" OR worker* OR "human exposure" OR "occupational exposure" OR "occupational exposure" [MeSH Terms] OR "occupational settings" OR "industrial settings" OR "occupational hygiene" OR "industrial hygiene" OR "human biomonitoring" OR "biomonitoring" [MeSH Terms] OR "exposure monitoring" OR "exposure at the workplace" OR "workplace" OR "occupational hazard" OR "occupational health surveillance") AND ("hexavalent chromium" OR "Cr(VI)" OR "chromium" OR "chromate" OR "chromium (VI)") AND (nickel OR ni))	**250**
PAHs + Ni + Cr(VI)	("human monitoring" OR "human biological monitoring" OR "occupational monitoring" OR "occupational exposure" OR "biomarkers of exposure" OR worker* OR "human exposure" OR "occupational exposure" OR "occupational exposure" [MeSH Terms] OR "occupational settings" OR "industrial settings" OR "occupational hygiene" OR "industrial hygiene" OR "human biomonitoring" OR "biomonitoring" [MeSH Terms] OR "exposure monitoring" OR "exposure at the workplace" OR "workplace" OR "occupational hazard" OR "occupational health surveillance") AND (("Polycyclic aromatic hydrocarbons" OR "benzo(a)pyrene" OR pyrene* OR PAHs OR PAHs [MeSH Terms]) AND (nickel OR ni)) AND ("hexavalent chromium" OR "Cr(VI)" OR "chromium" OR "chromate" OR "chromium (VI)")	**21**
	**Total studies**	**356**
